# InterRNA: a database of base interactions in RNA structures

**DOI:** 10.1093/nar/gkv1186

**Published:** 2015-11-08

**Authors:** Sri Devan Appasamy, Hazrina Yusof Hamdani, Effirul Ikhwan Ramlan, Mohd Firdaus-Raih

**Affiliations:** 1 School of Biosciences and Biotechnology, Faculty of Science and Technology, Universiti Kebangsaan Malaysia, 43600 UKM Bangi, Selangor, Malaysia; 2 Department of Artificial Intelligence, Faculty of Computer Science and Information Technology, University of Malaya, Kuala Lumpur, Malaysia; 3 Institute of Systems Biology, Universiti Kebangsaan Malaysia, 43600 UKM Bangi, Selangor, Malaysia

## Abstract

A major component of RNA structure stabilization are the hydrogen bonded interactions between the base residues. The importance and biological relevance for large clusters of base interactions can be much more easily investigated when their occurrences have been systematically detected, catalogued and compared. In this paper, we describe the database InterRNA (INTERactions in RNA structures database—http://mfrlab.org/interrna/) that contains records of known RNA 3D motifs as well as records for clusters of bases that are interconnected by hydrogen bonds. The contents of the database were compiled from RNA structural annotations carried out by the NASSAM (http://mfrlab.org/grafss/nassam) and COGNAC (http://mfrlab.org/grafss/cognac) computer programs. An analysis of the database content and comparisons with the existing corpus of knowledge regarding RNA 3D motifs clearly show that InterRNA is able to provide an extension of the annotations for known motifs as well as able to provide novel interactions for further investigations.

## INTRODUCTION

For the first two decades of the Protein Data Bank's (PDB) existence, the number of RNA structure entries did not exceed 1000 structures (1). However, in the past 15 years, numerous RNA structures have been determined including the large and small ribosomal subunits (2–4), riboswitches (5–7), and both group I and group II introns (8,9). This progress is in part due to the advances achieved in the field of X-ray crystallography and electron microscopy coupled with interest generated by the increasing availability and functional relevance of RNA molecules.

As a consequence, knowledge regarding the complexity and variety of RNA tertiary structure has greatly expanded. Additional structural studies have shown that these molecules consist of recurrent structural elements or motifs that are largely formed by non-canonical pairings in addition to the standard Watson–Crick pairings (10). These structural motifs are characterized at both the secondary and tertiary levels of RNA structure. Examples of secondary structural motifs include the various hairpin, internal and junction loops, whereas tertiary structural motifs include interactions such as the A-minor motifs, ribose-zippers, loop-receptor motifs and several others (11,12). These interactions are largely involved in stabilizing the tertiary structure of RNA (12,13) while some have been implicated in functional roles (14,15). Apart from these motifs, other examples of RNA tertiary interactions such as base interaction clusters are also present in RNA molecules (10,16). It is important to differentiate the terminology used where the term motif refers specifically to recurring tertiary arrangements or interactions of the RNA bases. Therefore specific base arrangements or base interactions need not necessarily be motifs. A base interaction cluster refers specifically to a group of bases where at least one hydrogen bond connects one base to another continuously. Again, a specific base interaction need not necessarily be motifs.

At present, several databases that contain annotation of RNA structural interactions exist. NCIR is a database of non-canonical interactions found in RNA structures and is compiled through a literature search (10). For each base pair type, NCIR provides information about the sequence and structure context in which the base pair type has been found. SCOR is a structural database that consists of internal and hairpin loops (17). Its classification scheme is based on a directed acyclic graph, allowing a node to have multiple parents. Another resource, the RNAJunction database, contains structure and sequence information for RNA junctions, kissing loops, internal loops and bulges (18). One interesting search capability of this database is the use of inter-helical angles for identifying RNA junctions. The kink-turn database provides a list of putative and known kink-turns in RNA structures with additional features that allow users to visualize and align the 3D structure of kink-turns (19). The RNA 3D Motif Atlas database contains internal and hairpin loop RNA 3D motifs that were automatically classified (20). Finally, the RNA Bricks database contains records of recurrent RNA 3D motifs and their contacts with other RNA motifs, proteins, metal ions, water molecules or small molecule ligands (21).

Many of the existing databases are dedicated for a specific group of RNA structural motifs (such as the kink turn database) and do not represent the diverse repertoire of RNA structural motifs that are present in an RNA structure. Several of these databases are also no longer updated regularly and several are also no longer accessible on the web. Furthermore, none of these databases specifically provides annotation on RNA base interaction clusters or a mapping of the interactions between the bases in forming specific 3D arrangements. These interactions can potentially act as structural motifs and therefore, have functional or structural stabilization roles. In order to address the limitations of currently available resources, we have developed a database called InterRNA (Interactions in RNA nucleotides database). This database is a repository of 3D base arrangements and clusters of hydrogen bonded base interactions that were the result of annotations by the NASSAM (22,23) and COGNAC (16) computer programs.

## METHODS AND DATABASE ACCESS

### Data set and annotation methods

The current data set for InterRNA consists of 2192 RNA structure files downloaded from the PDB using the following criteria: (i) contains RNA chain, (ii) solved by either X-ray crystallography or electron microscopy and (iii) having a structure resolution equal or better than 4 Angstroms (≥ 4.0 Å). InterRNA's data set is updated on a monthly basis. The downloaded structures were then annotated for specific base arrangement patterns of known tertiary motifs using the program NASSAM and for clusters of three to six bases that are interconnected by hydrogen bonds using the program COGNAC. NASSAM is a graph theoretical algorithm that is able to search an input structure containing RNA chains against a database of base arrangements (22,23). Due to the requirement of needing prior knowledge regarding a motif for the NASSAM program, a second algorithm, COGNAC (16), was used to identify patterns of interacting bases without needing prior knowledge of their spatial arrangement to each other. Both these programs and their usage for the structural annotation of 3D base arrangements have been previously described in detail by Harrison *et al*. and Hamdani *et al*. for NASSAM (22,23) and Firdaus-Raih *et al*. for COGNAC (16).

### Automated Annotation of RNA structural motifs using NASSAM

The NASSAM computer program was used to annotate: (i) Type I and Type II A-minor motifs (24), (ii) base-triples (25,26), (iii) tetraloops (27–29), (iv) ribose-zippers (30) and (v) T-loops (31). The NASSAM retrieved arrangements were further screened using motif specific filters to prevent contamination of the database with false positives. In the case of base-triples, only NASSAM hits that were planar interactions consisting of at least two hydrogen bond interactions per base pair were included in the database. The Type I and Type II A-minor motifs were screened based on base-backbone hydrogen bonding patterns as defined by Nissen *et al*. (24). For ribose-zippers, only NASSAM hits that consisted of two consecutive O2’-O2’ hydrogen bonds between the chain segments were included in the database (30). Finally, tetraloops and T-loops were further filtered based on the number of consecutive nucleotides forming the hairpin loop (Tetraloop = 4, T-loop = 5).

### Manual curation of NASSAM annotated RNA structural motifs

The InterRNA database also contains a set of manually curated annotations for sixty four RNA structures that was originally a validated motifs data set used for the development of the NASSAM computer program. NASSAM pseudo-atom vectors were handcrafted for each motif, then used as queries to search the test data set of 64 structures. The structures in this data set were selected from the work reported by Xin *et al*. (31 structures) and 33 additional structures (mainly riboswitches and ribozymes) that were selected from literature. The hits were then visually examined to confirm whether they corresponded to the query or were false positives. Hits that were confirmed to be correct by this visual curation process were collected and pooled together into the manually curated InterRNA data set. Some types of motifs, mainly the loop types, are also not very amenable to automatic annotation by the NASSAM program because very strict filters had to be enforced thus resulting in many valid motifs being excluded. These leftover data that were annotated by NASSAM but screened out by additional filters are also visually examined and if confirmed as a valid motif, are also added to the manually curated data set section of the database.

### Annotation of hydrogen bonded base interaction clusters

**Figure 1.**
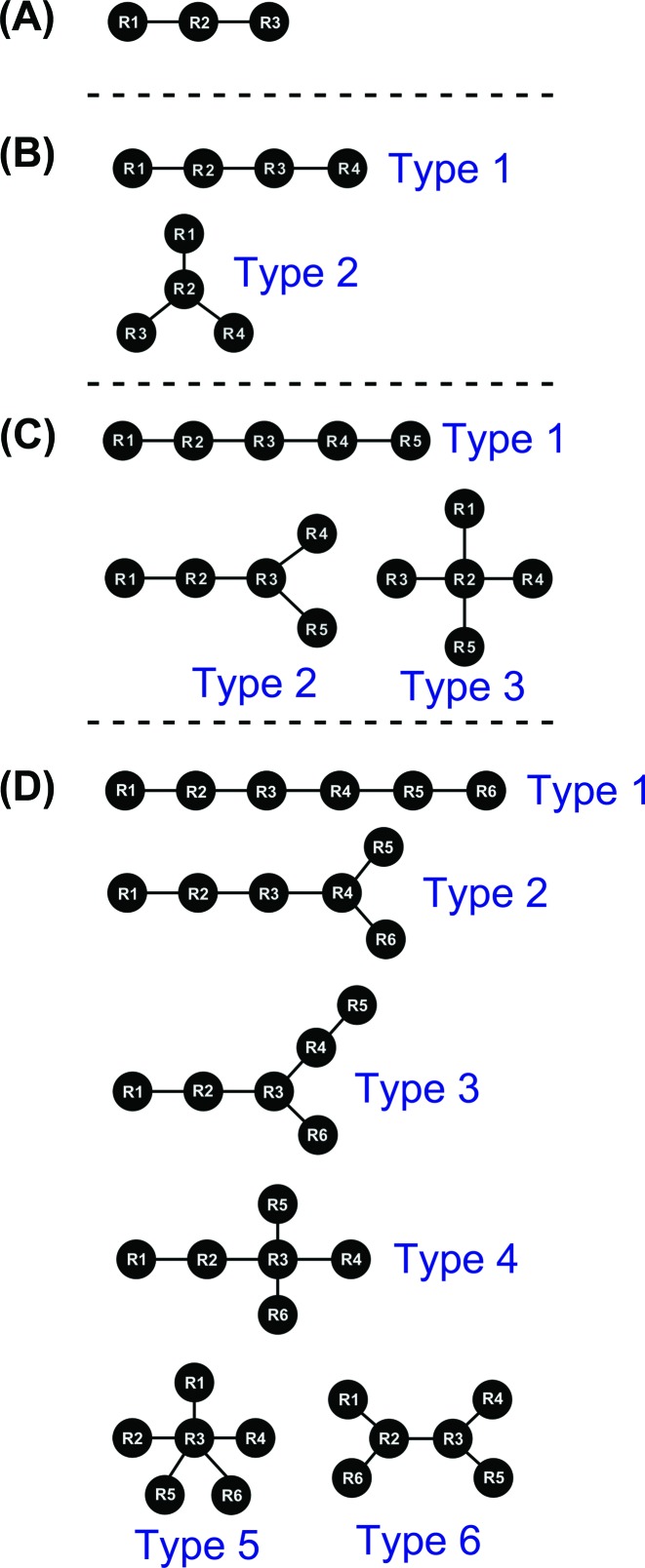
Tree graphs used by COGNAC to represent hydrogen bond connected base interactions where R denotes a base residue position (node) and the edges of the graphs are the lines connecting the nodes for (**A**) triples, (**B**) quadruples, (**C**) quintuples and (**D**) sextuples.

Hydrogen bonding data were first generated for all structures in the data set as described by Firdaus-Raih *et al*. (16). The COGNAC computer program was then used to identify small networks of base interactions that were represented by tree graphs where each base has at least one hydrogen bond connection as part of a network in a cluster of three to six bases (16) (Figure 1).

### Database access and interface

**Figure 2.**
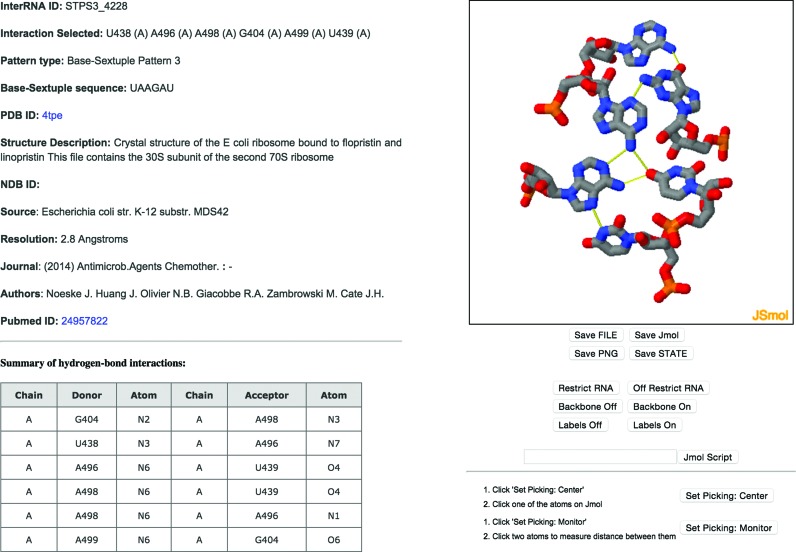
Example of the user interface displaying an InterRNA database record; the JSMol panel enables browser embedded molecular visualization of the entry which in this example is a sextuple type 3 pattern in a generally planar arrangement. A summary of the hydrogen bonds involved in the interaction is also provided in the lower left panel.

The InterRNA database is accessible via the URL http://mfrlab.org/interrna/ without any login requirements. Users have the choice of three searching methods: (i) an option to select a pre-determined set of arrangements and motifs; (ii) an option to search for base interaction annotations in a specific PDB structure using a PDB ID query or by using the unique database identifier; and (iii) a keyword search option. The specific base interactions and arrangements for all database entries can be visualized using the JSmol applet (Figure 2). Each entry is also pre-set to display any hydrogen bonds present for a specific interaction (Figure 2).

## CONTENT OVERVIEW AND DISCUSSION

Annotations by the NASSAM program are dependent on prior knowledge of a tertiary motif. The InterRNA database contains additional patterns for base arrangements that have been reported in the literature but were not originally part of the NASSAM server reported by Hamdani *et al*. (22). In order to demonstrate the value and utility of the InterRNA database compared to existing resources, we surveyed the content of several similar databases and compared these to the content available in InterRNA (Table 1). The comparison carried out shows that InterRNA does indeed fill a gap in the knowledge by providing an accounting of arrangements and motifs that are not covered by the other databases, such as the ribose zippers and Type I A-minor, Type II A-minor, Type II G-minor and the COGNAC annotated base interactions catalog. The database also extends the number of records for known arrangements and motifs that although are covered by other databases, do not have the extent of coverage provided by the InterRNA database. In the case of motifs that are not as easily computationally annotated without human intervention, such as several types of loops and turns, other databases fare better (Table 1). Although the NASSAM program is able to carry out such annotations, those motifs require additional visual inspection to determine the validity of the NASSAM retrieval. In this paper, we discuss some examples of how InterRNA provides a more extensive coverage of known motifs as well as an inventory of arrangements that are not available in other resources. Nevertheless, it is clear that the currently available databases complement each other due to the lack of a one-stop resource that is able to provide a complete accounting of all possible RNA tertiary motifs and base arrangement possibilities. An accounting of all the motifs and interactions that are available in the database is provided via a statistics page in the database's web interface.

**Table 1.** Comparison of content for similarly intended databases and web accessible resources involving base motifs in RNA structures

	NCIR	RNAJunction	RNA 3D Motif Atlas	RNA Bricks	InterRNA
Base pair	Yes	No	Yes	No	Yes
Base interaction clusters ranging from triples to sextuples	Yes. Contains base-triples, quadruples and quintuples	No	Yes. Only contains base-triples via RNA Triple Database	No	Yes. Contains base-triples, quadruples, quintuples and sextuples
Ribose-zippers	No	No	No	No	Yes
A/G-minor motifs	No	No	No	No	Yes
Hairpin Loop	No	No	Yes. Contains tetraloop (GNRA) & T-loop	Yes. Displays all the loops based on PDB ID. No classification is provided for the loops	Yes. Contains tetraloops (GNRA, UNCG & CUUG) and T-loops
Internal Loop	No	No	Yes. Contains C-loop, kink-turn, sarcin-ricin, triple sheared and double sheared	Yes. Displays all the loops based on PDB ID. No classification is provided for the loops	Yes. Only contains kink-turn (in the manually curated data set)
Junction Loop	No	Yes	No	Yes. Displays all the loops based on PDB ID. No classification is provided for the loops	No
Kissing Loop	No	Yes	No	No	No
Stems	No	No	No	Yes. Displays the stems based on PDB ID	No

### A catalog of the type I A-minor, type II A-minor and type II G-minor motifs

Previous studies have shown that the A-minor motifs and ribose-zippers are the two most abundant tertiary motifs in RNA structures (12,14,24,30). However, these studies have largely been restricted to specific groups of RNA structures, or in a data set of non-redundant RNA structures. As a result, there is no complete inventory available for A-minors and ribose-zippers in all high-resolution RNA structures. For the InterRNA database, only the Type I and Type II A-minor motifs were annotated by NASSAM because the Type 0 and Type III A-minors are neither particularly A-specific nor are they selective with regard to the receptor base-pair thus making the screening process of NASSAM retrieved hits for the correct arrangements a challenging task. Therefore, these A-minor arrangements were not automatically annotated to maintain the accuracy and quality of the computationally annotated base interactions in InterRNA. Apart from the standard A-minor motifs, InterRNA also has annotation records of the Type II G-minor motifs where the guanosine interacts with the minor-groove of a Watson–Crick receptor base-pair instead of an adenosine.

### InterRNA is the first online database containing ribose zipper motifs

The InterRNA database also provides annotation for six different types of ribose zippers: i) canonical ribose-zipper, ii) cis ribose-zipper, iii) naked ribose-zipper, iv) pseudo cis ribose-zipper, v) reverse single ribose-zipper and vi) single ribose-zipper and is the first and only resource to catalog these motifs (Table 1). Canonical ribose-zippers form the bulk of ribose-zippers annotated in RNA structures followed by single ribose-zippers. The two most common sequences for canonical ribose zippers are CC/AA and CU/AA respectively accounting for approximately 64% of all canonical ribose-zippers. The strong preference for the loop residues (**/RR) in a canonical ribose-zipper interaction to be purines is likely attributed to the tendency of these nucleotides to form specific A-minor contacts to link distant regions together (14,30).

### A catalog of base pairing diversity

The InterRNA database is also a repository that presents the diversity of base pairs and base triples that are able to occur. For the pairs annotated by COGNAC, any interaction of two bases, as long as it is connected by a hydrogen bond is considered a pairing. The canonical GC (CG) is the highest pair occurring in the base pairs and represented 46.4% of the overall base pairs annotation at the time of writing. The canonical AU (UA) makes up only 17.75% of the total base pairs annotated. The least number of pairings recorded was for the UC (CU) pair that represents 1.58% of the base pairs annotation. The same is true for the base triples annotation where we accept a single hydrogen bond connection between the bases in addition to accepting the non-planar orientation of the bases. This is different from the annotations of base triples by NASSAM that are restricted to planar interactions with at least two hydrogen bonds connecting each base pair in a triple.

As mentioned previously, there are numerous examples of hydrogen bond interconnected base clusters where some components of the interactions are no longer in the same plane and this is to be expected as the number of components in an interaction set gets larger. Nevertheless, the COGNAC annotations were also able to record large planar interactions such as the sextuple type 3 interaction found in the 16S rRNA *E. coli* subunit (Figure 2; PDB ID: 4tpe) (32). This interaction is located at helices 16 and 17 of 16S rRNA and is part of a five-way junction (33). This interaction is of interest because of the larger spatial requirement needed to form such a planar arrangement and thus can provide insights into potential interactions that can be used as base platforms in synthetic RNA structures.

### Complex clusters of base interactions

The current results of the COGNAC searches retrieved hits for hydrogen bond mediated base interactions involving pairs and triples as well as all types of quadruples, quintuples and sextuples. One example is the annotation of occurrences for base sextuple type 5 that requires a central base to interact with five other bases via hydrogen bonds. We observed that the central base position that is connected to the other five bases for all the eight hits retrieved is a guanine base. Although the sextuple type 5 interactions do not appear to be frequent, the records do provide insights into the possibility of complex clustering being able to occur, especially with regard to the flexibility of guanine in the central ‘anchor’ position. The quintuple type 3, in which one central base interacts with four other bases via hydrogen bonds, is similar to sextuple type 5 with one node less. This interaction accounts for approximately 79% of the examples recorded where the guanine base is the central base. The cytosine base as the central base only occurs 9.9% of the time, while the uracils and adenines make up the remaining 6.6% and 4.2%. Expectedly, guanine is the most likely candidate for the central role of such an interaction because of its high potential for hydrogen bonding with two hydrogen bond donor positions (atoms N1 and N2) and three acceptor positions (atoms O6, N3 and N7).

## SUMMARY

As more RNA structures are deposited in the PDB, value added information that could be extracted from the structural data with regard to the motifs and arrangements of the bases could be useful. For example if an investigator were to query whether a particular arrangement of three to six bases is present in any available structures, the InterRNA database would be able to provide the answer. The InterRNA database is also unique in being a repository for records of not only known motifs but various other types of base arrangements that are not necessarily classed as motifs. A comparative analysis of the base arrangements in different structures of the same molecule may offer insights into changes at the atomic level that contribute to a mechanistic effect of the RNA molecule's function.
